# Influenza A(H1N1)pdm09 But Not A(H3N2) Virus Infection Induces Durable Seroprotection: Results From the Ha Nam Cohort^[Author-notes jiaa293-FM2]^

**DOI:** 10.1093/infdis/jiaa293

**Published:** 2020-06-02

**Authors:** Le Nguyen Minh Hoa, Sheena G Sullivan, Le Quynh Mai, Arseniy Khvorov, Hoang Vu Mai Phuong, Nguyen Le Khanh Hang, Pham Quang Thai, Le Thi Thanh, Louise Carolan, Dang Duc Anh, Tran Nhu Duong, Juliet E Bryant, H Rogier van Doorn, Heiman F L Wertheim, Peter Horby, Annette Fox

**Affiliations:** Oxford University Clinical Research Unit and Wellcome Trust Major Overseas Programme, Hanoi, Viet Nam; WHO Collaborating Centre for Reference and Research on Influenza, Peter Doherty Institute for Infection and Immunity, Melbourne, Victoria, Australia; Doherty Department, University of Melbourne, Peter Doherty Institute for Infection and Immunity, Melbourne, Victoria, Australia; Fielding School of Public Health, University of California, Los Angeles, California, USA; National Institute of Hygiene and Epidemiology, Hanoi, Viet Nam; Doherty Department, University of Melbourne, Peter Doherty Institute for Infection and Immunity, Melbourne, Victoria, Australia; National Institute of Hygiene and Epidemiology, Hanoi, Viet Nam; National Institute of Hygiene and Epidemiology, Hanoi, Viet Nam; National Institute of Hygiene and Epidemiology, Hanoi, Viet Nam; National Institute of Hygiene and Epidemiology, Hanoi, Viet Nam; WHO Collaborating Centre for Reference and Research on Influenza, Peter Doherty Institute for Infection and Immunity, Melbourne, Victoria, Australia; National Institute of Hygiene and Epidemiology, Hanoi, Viet Nam; National Institute of Hygiene and Epidemiology, Hanoi, Viet Nam; Oxford University Clinical Research Unit and Wellcome Trust Major Overseas Programme, Hanoi, Viet Nam; Center for Tropical Medicine and Global Health, Nuffield Department of Clinical Medicine, University of Oxford, Oxford, UK; Oxford University Clinical Research Unit and Wellcome Trust Major Overseas Programme, Hanoi, Viet Nam; Center for Tropical Medicine and Global Health, Nuffield Department of Clinical Medicine, University of Oxford, Oxford, UK; Oxford University Clinical Research Unit and Wellcome Trust Major Overseas Programme, Hanoi, Viet Nam; Department of Medical Microbiology, Radboud University Medical Center, Nijmegen, Netherlands; Oxford University Clinical Research Unit and Wellcome Trust Major Overseas Programme, Hanoi, Viet Nam; Center for Tropical Medicine and Global Health, Nuffield Department of Clinical Medicine, University of Oxford, Oxford, UK; Oxford University Clinical Research Unit and Wellcome Trust Major Overseas Programme, Hanoi, Viet Nam; WHO Collaborating Centre for Reference and Research on Influenza, Peter Doherty Institute for Infection and Immunity, Melbourne, Victoria, Australia; Department of Microbiology and Immunology, University of Melbourne, Peter Doherty Institute for Infection and Immunity, Melbourne, Victoria, Australia

**Keywords:** influenza A virus, antibody, immunity, H1N1 subtype, H3N2 subtype, cohort studies

## Abstract

**Background:**

The extent to which influenza recurrence depends upon waning immunity from prior infection is undefined. We used antibody titers of Ha-Nam cohort participants to estimate protection curves and decay trajectories.

**Methods:**

Households (270) participated in influenza-like–illness (ILI) surveillance and provided blood at intervals spanning laboratory–confirmed virus transmission. Sera were tested in hemagglutination inhibition assay. Infection was defined as influenza virus-positive ILI and/or seroconversion. Median protective titers were estimated using scaled-logistic regression to model pretransmission titer against infection status in that season, limiting analysis to households with infection(s). Titers were modelled against month since infection using mixed-effects linear regression to estimate decay and when titers fell below protection thresholds.

**Results:**

From December 2008–2012, 295 and 314 participants were infected with H1N1pdm09-like and A/Perth/16/09-like (H3N2Pe09) viruses, respectively. The proportion protected rose more steeply with titer for H1N1pdm09 than for H3N2Pe09, and estimated 50% protection titers were 19.6 and 37.3, respectively. Postinfection titers started higher against H3N2Pe09 but decayed more steeply than against H1N1pdm09. Seroprotection was estimated to be sustained against H1N1pdm09 but to wane by 8-months for H3N2Pe09.

**Conclusions:**

Estimates indicate that infection induces durable seroprotection against H1N1pdm09 but not H3N2Pe09, which could in part account for the younger age of A(H1N1) versus A(H3N2) cases.

## Teaser

To estimate the duration of seroprotection following influenza infection we modelled the protective effects, and decay, of hemagglutination inhibiting antibodies. A(H1N1) but not A(H3N2) infection induced durable seroprotection, and accordingly A(H1N1) predominantly infected children whereas A(H3N2) infected all age groups.

The recurrence of influenza epidemics has largely been associated with virus antigenic drift and the ensuing accumulation of susceptible individuals [1–3]. Most notably, major A(H3N2) epidemics have coincided with large changes in the antigenic profile of circulating viruses [4]. Less is known about how the induction and maintenance of immunity via sustained antibody production impacts upon epidemic recurrence or the age structure of epidemics.

Hemagglutination inhibiting (HI) antibodies block virus attachment to host cells [5]. Accordingly, early challenge studies showed that the risk of infection among vaccinees whose postvaccination titers were at least 40 was reduced by 50% compared to vaccinees with lower titers [6]. Numerous subsequent studies demonstrated that relationships between HI titers and protection were heterogeneous and were modified by factors such as age and type of antigen exposure; 50% protection titers of HI antibodies have ranged from 8 to 260 [6–12] and tend to be lower for immunity induced by natural infection than by vaccination, particularly in older age groups [9, 12, 13], presumably because natural infection induces immune responses beyond HI antibodies.

**Figure 1.**
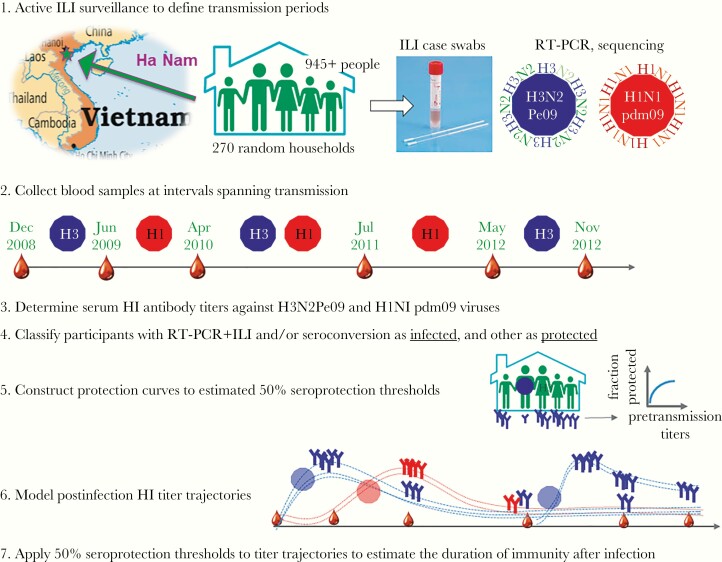
Study design. Diagrams depict protocols for selection and investigation of Ha Nam Cohort participants to detect H1N1pdm09 and H3N2Pe09 infections, and subsequently estimate HI titer protection thresholds, titer decay, and the duration seroprotection. Abbreviations: HI, hemagglutination inhibiting; ILI, influenza-like illness; RT-PCR, reverse transcription polymerase chain reaction.

HI titers are expected to peak within 1 month of influenza exposure and decline over the subsequent 6 to 12 months, reflecting expansion and contraction of short-lived antibody-secreting cells [14]. Thereafter, antibody titers typically remain stable [15, 16], depending upon the generation of long-lived antibody-secreting cells, which can persist for decades if B cells develop sufficient antigen affinity to compete for space within survival niches [14]. The majority of studies examining influenza HI titer dynamics involve vaccine recipients, some of which report rapid HI titer decline [16–18] and justify annual influenza vaccination [19]. Yet others report that titers remain above protective thresholds beyond a single influenza season [20–23], and that prior infection with related influenza viruses may promote antibody persistence after vaccination [24]. It is well established that influenza infection induces more sustained protection than vaccination [15, 25–28], suggesting that the magnitude or breadth of the antibody response may also be greater. Nevertheless, few studies examine titer trajectories post infection. Smith and Davies studied influenza A(H3N2) virus infections in boys attending boarding school and found that 8 of 13 boys who had been infected had more than a 2-fold drop in HI titers over 9 months [15]. More recently, Delabre et al found that titers against a seasonal A(H1N1) virus dropped less than 2-fold more than 2 years after presenting to health care services with confirmed influenza-like illness (ILI) [12]. However, a substantial proportion of infected people do not present to health care services [26, 29–31]. Titer decay in this group can be determined through cohort studies using seroconversion to detect mild and subclinical infections. In this study, data from a household influenza cohort in Ha Nam, Viet Nam were used to estimate HI titers associated with 50% protection against influenza A virus infections and to estimate postinfection titer trajectories (Figure 1). Seroprotection thresholds were then applied to postinfection titer trajectories to estimate the average duration of protection. Estimates for H3N2Pe09 and H1N1pdm09 were compared to investigate whether differences in the epidemiology of these viruses could be influenced by antibody effectiveness or maintenance.

## METHODS

### Participants

This study involved participants of the Ha Nam household cohort, who took part in active investigation to detect influenza infection as depicted in Figure 1. The cohort was established in December 2007 and has been described in detail elsewhere [11, 29]. In brief, approximately 1000 members of 270 randomly selected households participated in active surveillance for ILI, defined as fever with cough or sore throat. Pooled nose and throat swabs from ILI cases were assessed by reverse transcription polymerase chain reaction (RT-PCR) to detect influenza virus RNA [29]. Sera collected from available participants aged 5 years or older, at variable intervals spanning transmission periods, were assessed by HI assay with circulating viruses. This study includes the period between December 2008 and 2012 when H1N1pdm09-like and H3N2Pe09-like influenza viruses circulated. For the majority of participants, the last sample included in the analysis was collected in November 2012. This was extended to include samples collected in August 2013 and January 2014 for participants with illness confirmed by RT-PCR to be due to A(H3N2) virus infection to increase the sample size for this group.

### Ethics Statement

Written informed consent was obtained from participants or their parents/legal guardians. Approval to conduct the study was granted by the institutional review boards of the National Institute of Hygiene and Epidemiology, Vietnam and the Oxford Tropical Research Ethics Committee, University of Oxford, UK.

### Hemagglutination Inhibition Assays

Sera were diluted 2-fold from 1:10 to 1:1280 to titrate antibodies against H1N1pdm09 and H3N2Pe09-like viruses by HI assay with turkey erythrocytes. Titers were read as the reciprocal of the highest dilution that completely inhibited hemagglutination. The H3N2Pe09-like virus (A/Hanoi TX265/2009) was propagated from the swab of a case detected near Ha Noi during 2009, and had high HA identity to viruses isolated from participants during the same season [11]. The H1N1pdm09-like virus was provided by the Victorian Infectious Diseases Reference Laboratory, Melbourne, Australia, or was isolated from a cohort participant and propagated in MDCK cells. Sera from visit 3 were tested with both H1N1pdm09 antigens, and titers were highly correlated (*r* = 0.86, *P* < .001, based on Kendall *τ* measure of correlation). Nevertheless, participants whose sera indicated a ≥ 4-fold difference between antigens were excluded from the study (n = 9) to rule out interassay variability.

### Classifying Infections and Intervals From Blood Collection

Infection was defined as detection of viral RNA by RT-PCR in respiratory swabs, which were only collected if ILI was reported, and/or of seroconversion, that is a 4-fold or greater rise in HI titer between pre- and postseason serum samples.

H1N1pdm09 and H3N2Pe09 transmission times were determined via active ILI surveillance (Figure 1). For participants from households with infection detected by RT-PCR, the interval between each blood draw and the date of illness onset was calculated. For participants from households with infection detected by seroconversion alone, the date of infection was imputed as the midpoint of RT-PCR–confirmed case detection in the cohort during that transmission season (Table 1). If RT-PCR–confirmed infections were not detected between 2 blood sampling visits (n = 2; Table 1), the median date between visits was assigned as the date of transmission.

**Table 1.** Influenza Infections Detected and Sera Assessed by Subtype and Season

Sample	Season 1	Season 2	Season 3		Season 4		Season 5		Season 6^b^	Total H3N2, No.	Total H1N1, No.
	H3N2	H1N1	H3N2	H1N1	H3N2	H1N1	H3N2	H1N1	H3N2		
	**Season dates, d-mo-y**										
First ILI case	17-04-09	21-09-09	08-09-10	14-02-11	…	08-04-12	01-07-12	…	23-12-12		
Last ILI case	06-06-09	05-12-09	28-10-10	06-03-11	…	…	22-07-12	…	27-12-12		
Midtransmission date^**a**^	12-05-09	28-10-09	20-09-10	26-02-11	15-10-11	08-04-12	13-07-12	22-08-12	25-12-12		
Pretransmission bleed	12-12-08	06-06-09	03-04-10		08-07-11		19-05-12		24-11-12		
Posttransmission bleed	06-06-09	03-04-10	08-07-11		19-05-12		24-11-12		NA		
Paired sera, No.	503	549	555		571		620		NA		
ILI cases, No.	39	87	99		79		98		NA		
	**Influenza infections, No.**										
RT-PCR+ ILI	7	24	6	11	0	1	3	0	5	21	36
Seroconvert	107	132	104	107	32	17	63	28	4	311	284
Seroconvert with ILI^**c**^	6 ^1^	17 ^6^	5 ^1^	10	NA	0	3	NA	4^1^	18	27
Seroconvert w/o ILI	101	116	100	98	32	17	60	28	NA	293	259
Seroconvert or ILI	108	140	106	109	32	18	63	28	5	314	295
Reinfected	…	…	10	4	6	7	13	12	1	30	23
First infection	108	140	96	105	26	11	50	16	4	284	272
	**Decay, No. participants/visit (median postinfection day)** ^**d**^										
Postinfection visit 1	103 (25)	119 (157)	77 (291)	97 (132)	26 (158)	11 (41)	2 (131)^**b**^	…	3 (248)^**b**^	211	227
Postinfection visit 2	99 (326)	108 (618)	75 (607)	91 (448)	26 (347)	11 (230)	2 (485)^**b**^	…	3 (389)^**b**^	205	210
Postinfection visit 3	101 (787)	104 (934)	72 (796)	87 (637)	…	…	1 (557)^**b**^	…	…	174	191
Postinfection visit 4	95 (1103)	106 (1123)	4 (1082)	…	…	…	…	…	…	99	106
Postinfection visit 5	88 (1292)	…	3 (1219)	…	…	…	…	…	…	91	
Postinfection visit 6	6 (1572)	…	…	…	…	…	…	…	…	6	
Postinfection visit 7	5 (1712)	…	…	…	…	…	…	…	…	5	

Abbreviation: ILI, influenza-like illness; NA, not available (sera collected at the end of season 6 was not routinely assessed against H3N2Pe09); RT-PCR, reverse transcription polymerase chain reaction.

^a^The median transmission date was used if ≥ 2 ILI cases were detected, otherwise an individual ILI case date, or the mid-date between bleeds was used.

^b^Data were limited to participants who had confirmed H3N2-positive ILI, for whom we also tested sera collected in 2013 and 2014.

^c^Number who had RT-PCR-positive ILI but lacked paired sera required to detect seroconversion are shown in superscripts.

^d^Participants who had 2 + postinfection sera. Reinfected participants are included at their first infection season only.

### Analysis of HI Titer Data to Estimate Protective Thresholds and Titer Trajectories

Data cleaning was conducted in SAS for Windows v9.4 (SAS Institute), while all analyses were conducted in R v3.5.2. In all analyses, the midpoint log_2_ titer was used to overcome the downward bias associated with interval censoring in titer data [32, 33]. For example, a titer value of 20 equates to a midpoint value of 28.28.

Pretransmission HI titers from participants of households in which at least 1 influenza infection was identified were modelled against protection using a scaled logistic regression model. The model was fit using a Bayesian approach, with the prior mean and standard deviation of log HI titers selected to be broad but still reflect the expected distribution in the general population (Supplementary Material 2). The 95% credible intervals were obtained from bootstrap sampling.

Average antibody decay was modelled using log_2_ midpoint HI titers and months since infection. Only participants who showed evidence of seroconversion and who had at least 2 postinfection titer measurements were considered (Table 1). Models were constructed using linear regression with random intercept (ie, individual) and slope (ie, months since probable infection) to account for the repeated antibody measures and different starting titers for each individual. This type of model handles unbalanced data well (Supplementary Material 1). Various spline terms were fit to allow for changes in trajectory noted during initial inspection of the data. For H1N1pdm09, a natural cubic spline with knots at 9 and 21 months was used. For H3N2Pe09, knots were additionally placed at 33 and 45 months due to the longer period of observation. The first knot was placed at 9 months to coincide with previously published estimates for the lifespan of short-lived plasma cells [14]. An unstructured covariance structure was used. Model fit was assessed by Akaike information criterion.

The time until titers decayed below estimated seroprotection thresholds for H1N1pdm09 and H3N2Pe09 were estimated from the models. In sensitivity analyses, we also estimated when titers fell below 20, 40, and 100, because these are commonly used thresholds [8, 34]. Separate models were fitted for each subtype to determine the contribution of age on decay kinetics. Age was categorized as < 15, 15–49, and ≥ 50 years, referred to as children, adults, and older adults, respectively. Interaction terms were included to estimate whether the decay differed by age category. For sensitivity analysis, we excluded participants who showed evidence of reinfection, defined as a further 4-fold rise in titer after seroconversion. We also compared decay between infections causing seroconversion with and without RT-PCR–confirmed illness. Models were fit and predicted means estimated using the lme4 package in R [35]; 95% prediction intervals were estimated using a semiparametric bootstrap for mixed models (bootMer in the lme4 package).

## RESULTS

### Detection of Influenza A Infections

**Figure 2.**
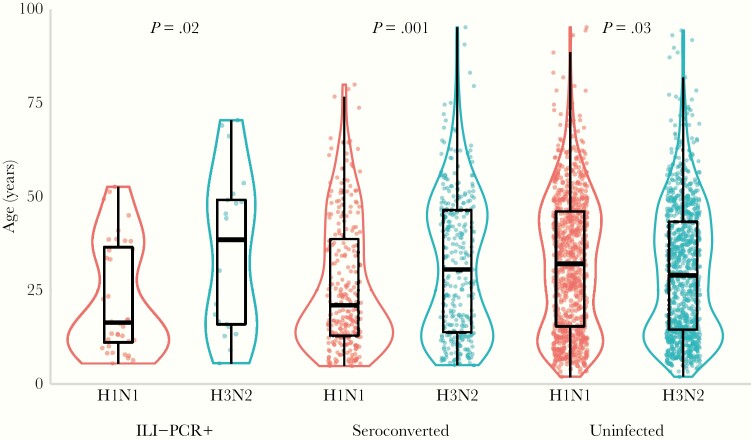
Age distribution of participants by infection status for each influenza A subtype from December 2008 to 2012. Dots represent individuals who were symptomatic and tested positive (ILI-PCR+), individuals without ILI symptoms but who seroconverted within a season (seroconverted), and individuals who neither seroconverted nor tested positive by PCR (uninfected). Violin plots show the distribution of individuals within age bands, while boxplots show the median and interquartile ranges. Mann-Whitney test *P* values compare participants with H1N1pdm09 versus H3N2Pe09 within each infection group. Abbreviations: ILI, influenza-like illness; PCR, polymerase chain reaction.

Table 1 summarizes influenza A infections detected as RT-PCR–confirmed illnesses and/or as seroconversions during the study period. H3N2Pe09-like virus transmission was first detected during April 2009, followed by H1N1pdm09-like virus in September 2009. Further periods of transmission were detected in 2010, 2011, and 2012. Viruses isolated in these periods were antigenically similar to those detected in 2009 (Supplementary Material 3). In total, 609 influenza A infections were detected, of which 552 (91.4%) were detected as seroconversion alone, and 57 as RT-PCR–confirmed illness either with seroconversion (n = 43), without seroconversion (n = 5), or without paired sera (n = 9). Of 272 individuals having H1N1pdm09-like virus infection 23 (8%) were reinfected, and of 284 individuals having H3N2Pe09-like virus infection 30 (11%) were reinfected. H1N1pdm09 infection and illness predominated among children and young adults whereas H3N2Pe09 was equally common in children and older age groups (Figure 2), suggesting that adults were relatively protected against H1N1pdm09 and in turn that the duration of immunity is longer.

### HI Protection Curves and 50% Protection Thresholds

**Figure 3.**
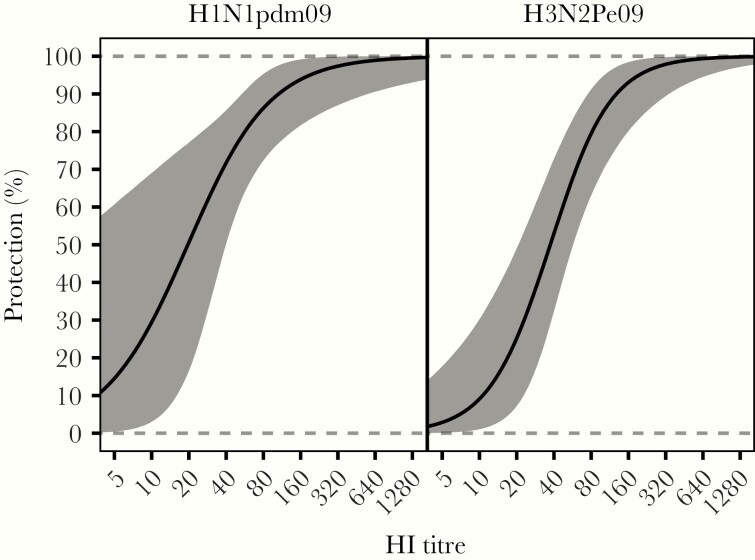
Hemagglutination inhibiting (HI) protection curves. Estimated probability of protection according to preseason HI titer for H1N1pdm09 (left) and H3N2pe09 (right). Graphs show the median of the posterior distribution (solid line), and the 95% credible intervals (CI; grey shading). Dashed lines show the prior distribution. For H1N1pdm09 the median protective titer was estimated at 20 (95% CI, 2–40), while for H3N2pe09 the median protective titer was 37 (95% CI, 21–57).

HI protection curves were generated using preseason titers of around 300 cases of each subtype detected during seasons in 2008–2012, together with over 400 preseason sera from noninfected members of their households. The distribution of preseason titers among infected and noninfected household members are summarized in Supplementary Table 2.1, which shows that the proportions infected decreased with increasing titer. Protection rose more steeply with titer for H1N1pdm09 than for H3N2Pe09 (Figure 3). Accordingly, estimated median 50% protection titers were 19.6 (95% credible interval, 2.03–39.7) for H1N1pdm09 and 37.3 (95% credible interval, 20.8–57.1) for H3N2Pe09. These median values were rounded to the nearest 10; that is a titer of 20 for H1N1pdm09 and a titer of 40 for H3N2Pe09 for calculation of the estimated time until geometric mean titers fell below protective levels (see section “Postinfection HI Titer Decay”).

### Postinfection HI Titer Decay

Titer decay was estimated for participants who had influenza A infection resulting in seroconversion and who had titers determined on at least 2 postinfection visits (Table 1). This included 211 participants with H3N2Pe09 infection and 236 with H1Npdm09 infection. However, the final analysis included 227 H1N1pdm09 cases; 9 were excluded due to discrepant HI titer results between the H1N1pdm09 antigens used for visits 1–3 versus visits 3–6. Among the participants assessed, 72 had infections with both subtypes, 15 had repeat H1N1pdm09 infection, and 27 had repeat H3N2Pe09 infection. Titer decay models were primarily based on 791 and 734 postinfection titers for H3N2P3e09 and H1N1pdm09, respectively, measured at intervals ranging from approximately 1 to 57 months post infection (Table 1).

**Figure 4.**
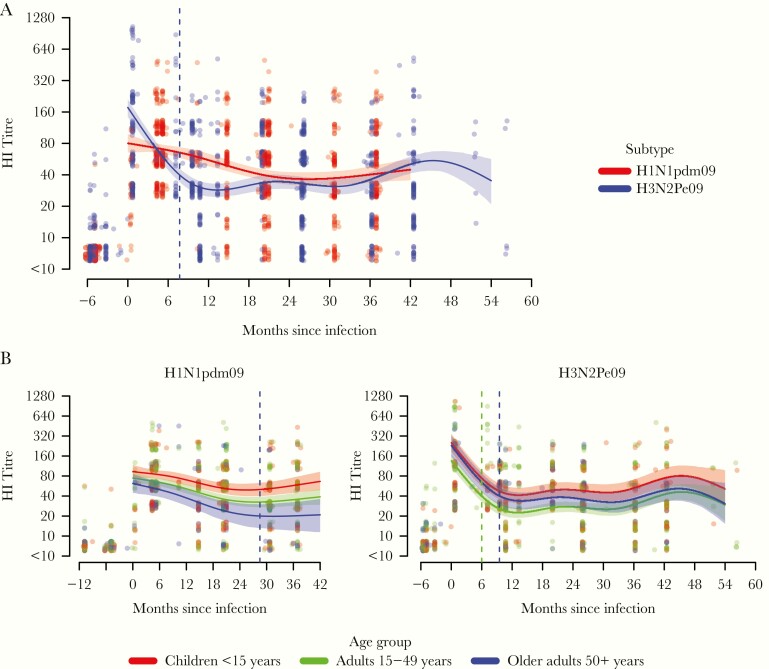
Estimated hemagglutination inhibiting (HI) antibody titer trajectories following H1N1pdm09 or H3N2Pe009 infection. *A*, The estimated decay modelled by subtype. *B*, Decay modelled by age group separately for H1N1pdm09 (left) and H3N2Pe09 (right). Dots indicate individual titers and are density-colored and jittered to permit visualization of the number of observations at each point. Lines show predicted mean titers and shaded areas show 95% prediction intervals. Dashed lines indicate the point at which the predicted means fell below the threshold for protection (a titer of 20 for H1N1pdm09 or 40 for H3N2Pe09).

Estimated mean postinfection titers were initially higher for H3N2Pe09 than for H1N1pdm09 (Figure 4A and Table 2), whereas fitted titer trajectories declined more steeply for H3N2Pe09 than for H1N1pdm09, particularly in the first year after infection (Figure 4A). There was a 6-fold decline in predicted mean titers over 12 months for H3N2Pe09 but only a 1.4-fold decline for H1N1pdm09 (Table 2). Correspondingly, predicted mean titers remained above the 50% protection threshold of 20 for the 36 months of follow-up for H1N1pdm09 but not for H3N2Pe09, which decayed below its protective titer of 40 by 8 months (Table 2 and Figure 4A). When applying the higher H3N2Pe09 threshold of 40 to the H1N1pdm09 data, mean predicted titers fell below protective levels 21 months after infection. In contrast, the mean predicted titer was never higher than 100 for H1N1pdm09 but remained higher than 100 for at least 2.5 months for H3N2Pe09 (Table 3).

**Table 2.** Predicted Geometric Mean Titers, 95% Prediction Intervals, and the Absolute and Relative Change in Titer at Baseline^a^ and Selected Times Post Infection, by Age Group and Overall

Time Post Infection, mo	Children <15 y			Adults 15–49 y			Older Adults 50+ y			Overall		
	Predicted Mean (95% PI)	Δ abs	Δ rel	Predicted Mean (95% PI)	Δ abs	Δ rel	Predicted Mean (95% PI)	Δ abs	Δ rel	Predicted Mean (95% PI)	Δ abs	Δ rel
H1N1pdm09^b^												
Baseline	92.8 (71.7–116.9)	0	1	74.5 (58.3–90.6)	0	1	61.5 (43.7–86.5)	0	1	79.9 (64–99.7)	0	1
6	83.7 (72.8–98.3)	−9.1	1.1	64.2 (56.2–72.9)	−10.3	1.2	49.8 (37.0–67.4)	−11.7	1.2	68.6 (62.4–75.4)	−11.3	1.2
12	69.9 (58.7–84.3)	−22.9	1.3	51.3 (43.7–60.4)	−23.2	1.5	37.4 (27.0–52.0)	−24	1.6	55.2 (48.3–62.9)	−24.8	1.4
18	55.8 (47.5–67.5)	−37	1.7	39.1 (33.6–45.4)	−35.4	1.9	26.8 (19.1–37.0)	−34.6	2.3	42.8 (38.4–47.9)	−37.2	1.9
24	49.5 (40.7–60.7)	−43.3	1.9	33.2 (27.6–39)	−41.3	2.2	21.4 (14.6–31.0)	−40.1	2.9	36.8 (32.3–42)	−43.1	2.2
36	57.5 (44.9–73.8)	−35.3	1.6	35.2 (28.1–43.9)	−39.3	2.1	20.1 (11.8–33.8)	−41.4	3.1	40.1 (33.7–47)	−39.8	2
H3N2Pe09^c^												
Baseline	252.5 (188.6–333.7)	0	1	135 (108 170.6)	0	1	229.8 (163.4–326.7)	0	1	176.5 (147.1–217.4)	0	1
6	73.9 (57.1–96.2)	−178.5	3.4	39.9 (33.3–47.2)	−95.1	3.4	64.3 (47.2–87.8)	−165.6	3.6	51.5 (44.7–59.3)	−125	3.4
12	41.7 (33.0–54.2)	−210.7	6	22.7 (19–26.9)	−112.2	5.9	34.6 (25.7–46.7)	−195.2	6.6	29 (25.1–33.1)	−147.6	6.1
18	45.8 (36.5–59.5)	−206.6	5.5	25.2 (20.9–30.2)	−109.8	5.4	36.3 (27.1–49.2)	−193.5	6.3	31.7 (27.0–36.8)	−144.9	5.6
24	49.4 (39.1–63.5)	−203.1	5.1	27.4 (22.8–32.8)	−107.6	4.9	37.3 (28.2–50.7)	−192.5	6.2	34 (29.4–39.5)	−142.5	5.2
36	52.4 (39.7–70.3)	−200	4.8	29.6 (23.8–37.3)	−105.3	4.6	36.2 (25.3–50.3)	−193.7	6.4	35.9 (30.5–43.1)	−140.7	4.9

Δ titer compares the titer at each time with the baseline titer on either the absolute scale (abs) or relative scale (rel); Baseline titer is based on the blood sample taken closest to infection.

Abbreviations: abs, absolute; PI, prediction interval; rel, relative.

^a^Baseline refers to the estimated titer at the time of infection. The model does not include preinfection titers such that baseline values are indicative of estimated early postinfection titers.

^b^H1N1pdm09: children <15 y, n = 82; adults 15–49 y, n = 122; older adults 50+ y, n = 23; overall n = 227.

^c^H3N2Pe09: children <15 y, n = 56; adults 15–49 y, n = 115; older adults 50+ y, n = 40; overall, n = 211.

**Table 3.** Sensitivity Analysis Showing the Time at Which Predicted Mean Titers Fell Below Selected Thresholds for Protection, by Age Group

Age Group	Months after Infection					
	Threshold Titer = 20		Threshold Titer = 40		Threshold Titer = 100	
	H1	H3	H1	H3	H1	H3
Overall			21	7.7	0	2.6
Children <15 y					0	4.4
Adults 15–49 y			17.5	6	0	1.4
Elderly 50+ y	28.5		10.8	9.5	0	3.8

Empty cells indicate that the mean predicted titer never fell below the threshold; 0 indicates that the mean predicted titer never exceeded the threshold.

### Effect of Age on HI Titer Decay

Estimated mean H1N1pdm09 titers remained above the threshold for protection of 20 for children (<15 years) and adults (15–49 years), but fell below the threshold by 28.5 months for older adults (≥50 years; Figure 4B and Table 3). This trend was consistent with the somewhat steeper H1N1pdm09 titer decline over 24 months in older adults (2.9-fold) compared to adults (2.2-fold) and children (1.9-fold) (Table 3). We also considered higher thresholds for protection (Table 3). At a threshold of 40, titers waned within 18 months for adults and older adults, and did not exceed a threshold of 100 for any age group.

Estimated mean H3N2Pe09 titers remained above the threshold for protection of 40 for children but fell below the threshold by 6 months for adults and 9.5 months for older adults (Figure 4B and Table 3). This trend reflected baseline H3N2P309 titers, which were almost 2-fold higher for children than for adults (Table 3), and a decline in titers that was slightly steeper at 12 months for older adults (relative change in titer of 6.6) compared with children (relative change of 6; Table 3 and Figure 4B). When considering the higher threshold of 100, titers waned by 4.4, 1.4, and 3.8 months for children, adults, and older adults, respectively (Table 3).

### Effect of Excluding Participants With Evidence of Reinfection on Postinfection Titer Decay

Forty-two participants showed evidence of reinfection, as indicated by a second 4-fold rise in titer (Supplementary Material 4). When compared with the rest of the cohort, these participants were similar in terms of observed preinfection HI titer, observed starting HI titer, and age. When these participants were excluded from the models, estimated H1N1pdm09 titers did not cross the protection threshold for at least 3 years, while H3N2Pe09 titer decreased below its threshold by 8.1 months.

### Titer Decay After Influenza Infection With Versus Without RT-PCR–Confirmed Illness

Titer decay was modelled separately for participants whose influenza infection caused seroconversion with and without RT-PCR–confirmed illness (Supplementary Material 5). Preinfection titers were similar for these groups, but participants who had H1N1pdm09 infection causing RT-PCR–confirmed illness were about 5 years younger than those who had infection detected by seroconversion alone. By contrast, participants who had H3N2Pe09 infection causing RT-PCR–confirmed illness were about 6 years older than those with seroconversion alone. Participants who had RT-PCR–confirmed infection had higher mean starting titers, but also had higher variance. Thereafter titer trajectories were similar for those infected with and without RT-PCR–confirmed illness.

## Discussion

This study estimated HI protection titers and postinfection titer decay among people aged 5–95 years who had never received influenza vaccine, and who were closely monitored to detect influenza infection, including that which caused seroconversion without reported ILI. Estimates were combined to determine if and when immunity induced by H3N2Pe09 or H1N1pdm09 infection wanes such that reinfection could occur independent of virus antigenic drift. Study estimates suggest that on average seroprotection against H3N2Pe09 persists for around 8 months, whereas seroprotection against H1N1pdm09 persists for at least 3 years. This in part reflected high protection rates at low titers for H1N1pdm09 compared to H3N2Pe09, but also less titer decay for H1N1pdm09 than for H3N2Pe09.

Differences in the estimated duration of immunity for H1N1pdm09 versus H3N2Pe09 could in part account for differences in the ages of people infected. Participants with H1N1pdm09 infection, with or without RT-PCR–confirmed illness, were younger than those with H3N2Pe09 infection. Similarly, studies conducted during periods when seasonal A(H1N1) and A(H3N2) viruses circulated found that A(H1N1) predominates in children whereas A(H3N2) affects all age groups [11, 29, 36, 37]. It may be expected that infections that induce durable immunity will be concentrated in children whereas infections that induce less durable immunity will recur throughout life such that infections will be dispersed across age groups. However, the extent to which age trends for H1N1pdm09 depend upon immunity from HI antibodies is debatable [11, 36, 38, 39], particularly during the first pandemic wave when few people had H1N1pdm09-reactive antibodies detected in prepandemic sera [11]. This was even observed for cohort participants born before 1957 when circulating A(H1N1) viruses were relatively similar to H1N1pdm09 [11]. Moreover, protection against A(H1N1) can increase with age and with prior infection independent of HI antibody detection, suggesting that non-HI antibodies, that recognize more conserved epitopes in HA and NA and have been associated with protection (reviewed in [40, 41]), may contribute more to protection against A(H1N1) than against A(H3N2) [11, 36]. A contribution of non-HI antibodies could also account for the relatively high levels of protection against H1N1pdm09 when HI titers are low. In a recent study, longitudinal titers from a cohort in Hong Kong were fitted to mechanistic models to dissect the dynamics of immunity following infection. These models indicate similar trends for the duration of immunity against H1N1 versus H3N2, and indicate that HI antibodies provide most of the protection detected in children but little of the protection detected in adults after infection, particularly for H1N1pdm09 [39].

Estimated postinfection titers were initially higher for H3N2Pe09 than for H1N1pdm09, but decay trajectories were much steeper for H3N2Pe09, consistent with results from the study in Hong Kong [39]. Petrie and colleagues also reported steep H3N2 antibody titer decline, which became steeper as peak titers increased [42]. H1N1pdm09 titer trajectories estimated from a study in Singapore were somewhat steeper than found here, potentially reflecting the older age (25–75 years) of participants in that study [43]. The duration of seroprotection varied with age in a subtype-dependent manner, being shortest amongst adults for H3N2Pe09 and shortest amongst older adults for H1N1pdm09. While age effects for H3N2Pe09 reflected differences in starting titers, age effects for H1N1pdm09 reflected steeper titer decline amongst older adults compared to children. Differences between H3N2Pe09 and H1N1pdm09 starting titers and trajectories could reflect the presence of memory B cells reactive with HI epitopes for H3N2Pe09 but not H1N1pdm09 [44–46]. In particular, we may expect that titer decline will be steeper if memory recall produces a larger short-lived plasma cell response that contracts exponentially [14].

The view that HI titer decay is more rapid in the elderly underpins advice to vaccinate no more than 4 months before expected influenza transmission [47]. In this study we detected some evidence of steeper titer decay amongst older adults compared with adults or children. However, the decline was only marginal at 6 months (and even at 12 months), which contradicts long-held wisdom about intraseasonal vaccine-induced antibody decline. In addition, the decline was more pronounced for H1N1 compared to H3N2, an observation that has been reported elsewhere [18, 48]. A review of vaccination studies in the elderly indicates that if seroconversion occurs, seroprotection rates remain high after 4 months, particularly against H3N2 [47].

Our analysis had several limitations. Data were not available for participants aged younger than 5 years. HI titer data is also limited by recording on an interval-censored scale, a limitation shared by all studies that rely on titer data. This means we know only the upper and lower limit, but not the exact amount of antibody needed for inhibition. In addition, titers are typically truncated by lower (in our case 10) and upper (in our case 1280) limits. Estimation from interval-censored, truncated data will tend to bias estimates downwards [32, 33]. We attempted to overcome this bias by using midpoint titers, which are, on average, closer to the true value than standard titers [32]. However, residual bias due to truncation of titers likely persists [8]. Sampling intervals added a further layer of interval censoring for those participants for whom infection was not detected as RT-PCR–confirmed illness. We attempted to overcome this problem by imputing the start date of infection for patients who only had serological evidence of infection. Finally, the variable contribution of data among individuals in the cohort may have affected model precision and could account for fluctuations in predicted titers. It is logistically challenging to collect samples at defined intervals post infection. In particular, for the majority of infections that do not cause ILI it would be necessary to collect swabs frequently to define when infection occurred. Nevertheless, future studies on antibody decay would benefit from more frequent and tailored sampling.

In conclusion, we generated HI protection and decay estimates that, when combined with information on virus antigenic change and antibody cross-recognition, can be used to better understand and forecast influenza epidemiology. The findings indicate that inferences about protection based on acute or peak titers should account for titer decay, which may differ with subtype and age. Further understanding of the cellular processes that shape titer trajectories, including the role of cross-reactive memory B cells, will benefit the development of epidemiological models and vaccines.

## Supplementary Data

Supplementary materials are available at *The Journal of Infectious Diseases* online. Consisting of data provided by the authors to benefit the reader, the posted materials are not copyedited and are the sole responsibility of the authors, so questions or comments should be addressed to the corresponding author.

jiaa293_suppl_Supplementary_Material

## Notes

***Acknowledgments***. We are grateful to the Ha Nam Preventive Medicine Centre and People’s Committees of Thanh Liem District for their support, and to the people of Thanh Ha Commune for participating in this study. We thank the Thanh Ha Commune health workers for their dedication to conducting active surveillance and cross-sectional surveys; the Ministry of Health of Vietnam for their continuing support of the research collaboration between the Oxford University Clinical Research Unit and the National Institute for Hygiene and Epidemiology; and Benjamin Cowling for his helpful comments on the initial draft and Sophia Ng for statistical advice.

***Financial support***. This work was supported by the Wellcome Trust United Kingdom (grant numbers 081613/Z/06/Z and 077078/Z/05/Z). The Melbourne WHO Collaborating Centre for Reference and Research on Influenza is supported by the Australian Government Department of Health.
