# Supplementary material for: Influenza A(H1N1)pdm09 But Not A(H3N2) Virus Infection Induces Durable Seroprotection: Results From the Ha Nam Cohort
Source: J Infect Dis. 2020 Jun 2;226(1):59–69. doi: 10.1093/infdis/jiaa293 (PMC9373157; doi:10.1093/infdis/jiaa293)
Supplement: jiaa293_suppl_Supplementary_Material [file jiaa293_suppl_supplementary_material.docx]

**Online supplement**

**Supplement 1. Comparison of the linear model for decay versus a polynomial model**

With polynomial (spline) models, the trajectory of decay is allowed to vary over time, which is more realistic than assuming a linear trajectory where decay would eventually reach a titre of 0. When a linear model is used, the slope appears steeper for H1pdm09, which hides the initial, more pronounced drop in titre observed for H3Pe09 (see Supplement Figure S5.1). After inspecting the raw data (Supplementary Table S5.1), and noting the fluctuations in titres in each 9-month period, we determined that a polynomial model would fit the data. This was confirmed by model diagnostics, such as AIC.


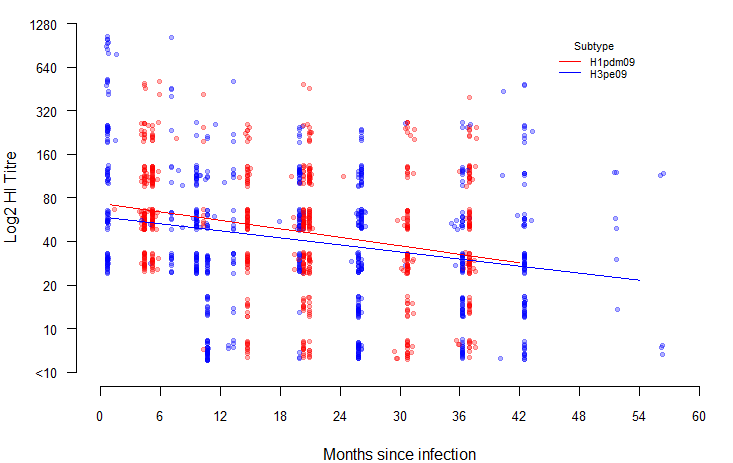


**Supplement Figure S1.1 HI titre decay when using a linear models to make predictions.**

**Supplementary Table S1.1 Raw means by months post-infection.** The difference between first and second visits is greater for H3N2Pe09 than for H1N1pdm09. Moreover, the raw means indicate decay is non-linear.

| Months post-infection | H1pdm09 | | H3pe09 | |
| --- | --- | --- | --- | --- |
|  | log_2_ titre | diff | log_2_ titre | diff |
| (0,9] | 6.16 | 0.00 | 6.45 | 0.00 |
| (9,18] | 5.54 | -0.62 | 4.97 | -1.48 |
| (18,27] | 5.44 | -0.09 | 5.21 | 0.24 |
| (27,36] | 4.93 | -0.52 | 5.82 | 0.61 |
| (36,45] | 5.35 | 0.43 | 4.77 | -1.05 |
| (45,54] |  |  | 5.66 | 0.88 |
| (54,63] |  |  | 4.42 | -1.23 |

**Supplement 2 – HI protection curves**

The scaled logit model was fit using a Bayesian approach:

$$Y_{i}\sim Bernoulli\left( p_{i} \right)$$

$$p_{i}=\frac{\lambda}{1+exp(\beta_{0}+\beta_{T}T_{i}}$$

$$T_{i}\sim N\left( \mu,\sigma^{2} \right)truncated\left( L_{i},H_{i} \right)$$

$$\mu\sim N\left( 2,2^{2} \right) \sigma\sim Exponential\left( rate=0.1 \right)$$

$$\lambda\sim Uniform\left( 0,1 \right)$$

$$\beta_{0}\sim N\left( -15,{10}^{2} \right) \beta_{T}\sim N\left( 5,5^{2} \right)$$

Where *i* is observation index, *Y_i_* is infection status (0 — infected (both symptomatic and asymptomatic), 1 — not infected), *T_i_* is true log HI titre, *L_i_* is the low bound of observed HI interval (e.g. for observation of 20 this is log(20)), *H_i_* is the high bound of observed HI interval (e.g. for observation of 20 this is log(40)).

The prior distributions for the mean and standard deviation of log HI titres were chosen to be broad but still somewhat reflect the expected distribution in the general population — mean high enough to allow a fair proportion (>20%) to have titres above detectable level (log(10)) and standard deviation low enough to not result in titres above highest detectable level (log(1280)) to be more prevalent than titres between log(640) and log(1280).

The prior distribution for the baseline risk parameter *λ* gives equal probability to all of its possible values. The prior distributions for the logistic curve parameters *β*_0_ and *β_T_* was chosen to be spread around all of their plausible values — *β*_0_ is likely below 0 (otherwise the probability of infection would be close to 0 and not change across the observed titre range) and not too low (e.g. a value below -30 would result in the probability of infection being equal to λ and not changing across the observed titre range); while *β_T_* is likely above 0 (since titres are protective) but not too high since it would result in a very steep curve (e.g. a titre of 20 may have the expected infection probability of *λ* while a titre of 40 has the expected infection probability of 0).

The sample was limited to only those households in which an infection has been detected (by either PCR or seroconversion). The data used are summarised in Table S2.1. The fitted infection and protection curves are shown in Figure S2.1.

**Supplementary Table S2.1.** Distribution of data used to estimate HI protection curves using pre-season titres for all members of households with at least one infected case.

| Subtype | Pre-season titre | Protected | (%) | Mean age | Infected | (%) | Mean age |
| --- | --- | --- | --- | --- | --- | --- | --- |
| H1N1pdm09 | <10 | 290 | 52% | 35.6 | 266 | 48% | 26.3 |
|  | 10 | 29 | 73% | 36.7 | 11 | 28% | 22.8 |
|  | 20 | 27 | 84% | 34.7 | 5 | 16% | 31.1 |
|  | 40 | 39 | 85% | 21.8 | 7 | 15% | 18.6 |
|  | 80 | 18 | 95% | 19.9 | 1 | 5% | 15.4 |
|  | 160 | 11 | 100% | 15.5 | 0 | 0% |  |
|  | 320 | 1 | 100% | 35.5 | 0 | 0% |  |
| H3N2pe09 | <10 | 264 | 52% | 36.5 | 243 | 48% | 34.9 |
|  | 10 | 67 | 57% | 29.8 | 51 | 43% | 25.7 |
|  | 20 | 74 | 76% | 24.5 | 23 | 24% | 21.8 |
|  | 40 | 39 | 78% | 20.9 | 11 | 22% | 24.1 |
|  | 80 | 29 | 91% | 13.4 | 3 | 9% | 27.8 |
|  | 160 | 17 | 100% | 22.7 | 0 | 0% |  |
|  | 320 | 2 | 100% | 35.6 | 0 | 0% |  |

**
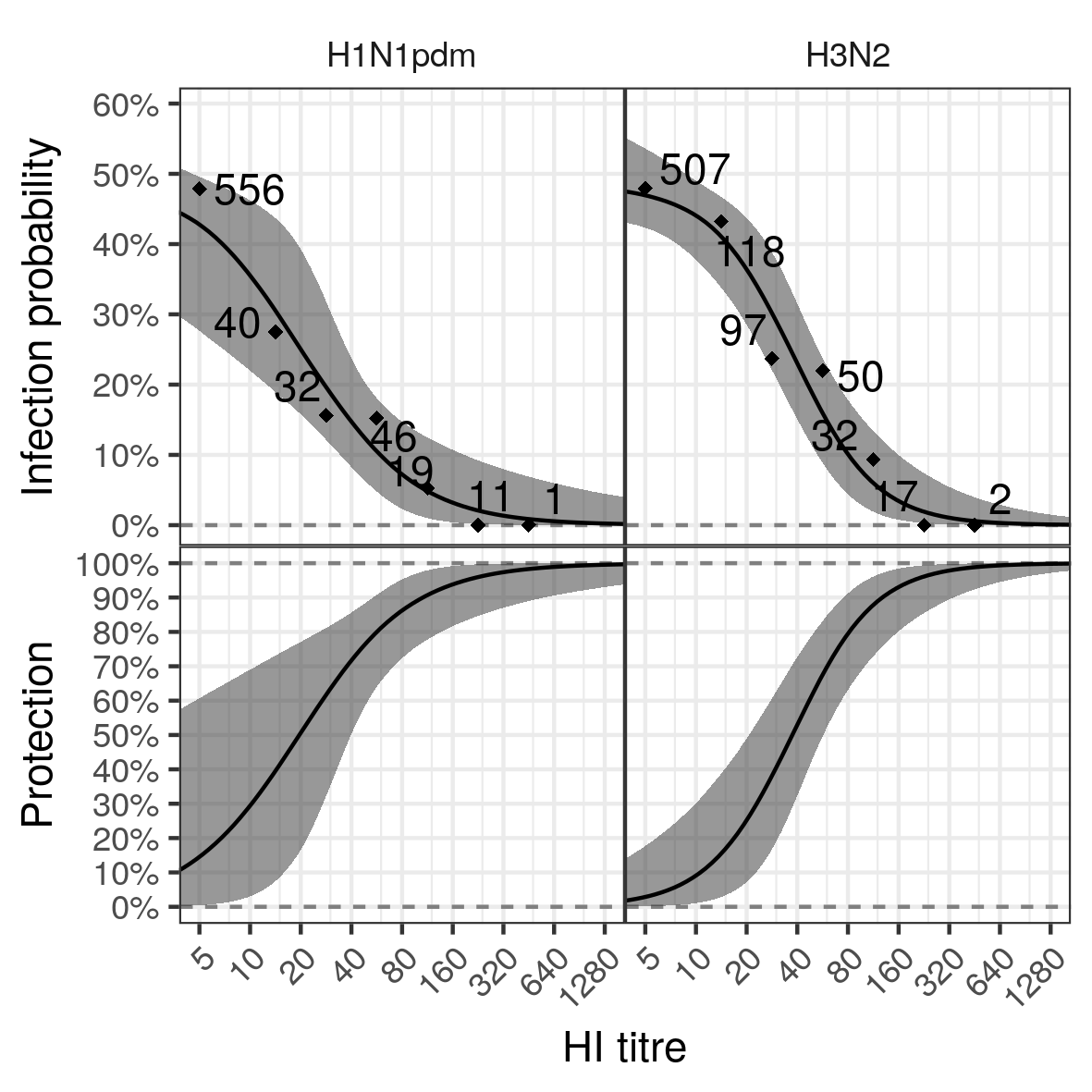
**

**Supplementary Figure 2.1.** Fitted infection (upper) and protection (lower) curves and their credible intervals from the scaled logit model. The solid line is the median of the posterior distribution. The shaded region is the 95% credible interval. The dashed line is the prior distribution, i.e. the bounds of the shaded region that would have been obtained if the data contained no information to estimate model parameters. The upper bound is above 90% and therefore not visible. The points shown in the upper panel are the infected proportions at the corresponding titre measurements. The numbers next to the points are the total sample size of the corresponding groups. Note that the titre measurements were shifted to midpoints of the corresponding intervals to better represent average underlying titres, e.g. the measurement of 20 was shifted to the log-scale midpoint between 20 and 40 (except the measurement of 5 which represents undetectable titres and the measurement of 1280 which represents titres above the highest detectable level).

**Supplement 3. Genetic and Antigenic Analysis of Influenza A viruses Isolated from Ha Nam Cohort** **Participant Swabs During the Period December 2008-2012**.

Viruses isolated from Ha Nam cohort paticipants were compared to vaccine strains by constructing phylogenetic trees (Supplementary Figure S3.1) and by performing serology with vaccine-strain antisera (Supplementary Table S3.2, Figure S3.2). A(H3N2) viruses isolated during 2009 were A/Perth/16/2009-like. Some genetic change was detected among viruses isolated later during the study period with genetic distance averaging 17.8 substitutions/1000 nucleotides. 2010 viruses contained amino acid subsitutions in antigenic sites E (K62E), A (K144N) and D (T212A), and 2012 viruses contained additional substitutions in antigenic sites E (H94Y), A (T131K), and B (A198S), but titres of A/Perth/16/09 antisera were equivalent against 2009, 2010 and 2012 viruses, as reported elsewhere [1] . However, titres of A/Victoria/361/2011 antisera were higher against 2012 viruses than against earlier viruses.

H1N1pdm09 viruses isolated from Ha Nam Cohort participants were A/California/07/2009-like Supplementary Figure S3.1). Viruses that circulatedin Viet Nam between 2010-2012 differed from 2009 viruses by 13 nucleotides/1000 on average, ranging from 5.8 to 18.8 nucleotides/1000. The main amino acid substitutions detected (S143G, S185T, A197T, E374K and S145N) have not been associated with antigenic change [2].

1. Eshaghi A, Duvvuri VR, Li A, et al. Genetic characterization of seasonal influenza A (H3N2) viruses in Ontario during 2010-2011 influenza season: high prevalence of mutations at antigenic sites. Influenza Other Respir Viruses **2014**; 8:250-7.

2. Barr IG, Russell C, Besselaar TG, et al. WHO recommendations for the viruses used in the 2013-2014 Northern Hemisphere influenza vaccine: Epidemiology, antigenic and genetic characteristics of influenza A(H1N1)pdm09, A(H3N2) and B influenza viruses collected from October 2012 to January 2013. Vaccine **2014**; 32:4713-25.


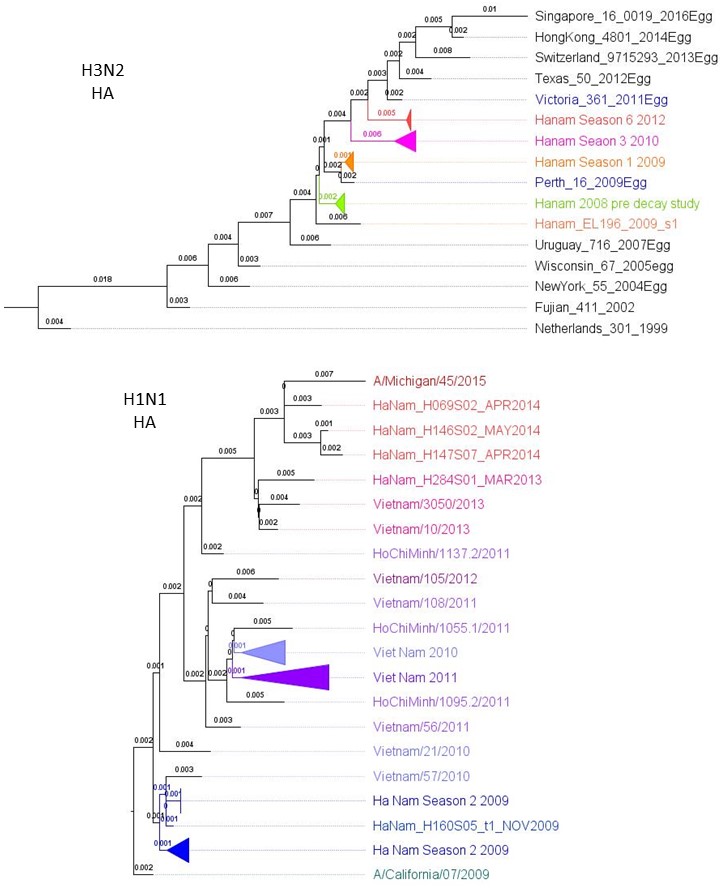


**Supplementary Figure S3.1.** HA gene sequences were determined for A(H3N2) and A(H1N1) viruses isolated from participants of the Ha Nam cohort who were infected during 2009 or adjacent years and compared to sequences of vaccine strains. A(H1N1) virus sequences included for 2010 and 2011 were from GISAID (Table S3.1). Nucelotide sequences were aligned using the MUSCLE algorithm in MegAlign Pro Version 13.0.0 (DNASTAR Inc). Branch lengths are shown.

**Supplementary Table S3.1 H1N1pdm09 sequences extracted from GISAID**

| **Designation** | **GISAID ID** | **Passage History** |
| --- | --- | --- |
| A/HoChiMinh/1192.3/2011 | EPI_ISL_163973 | clinical sample |
| A/HoChiMinh/1163.3/2011 | EPI_ISL_163972 | clinical sample |
| A/HoChiMinh/1155.3/2011 | EPI_ISL_163971 | clinical sample |
| A/HoChiMinh/1137.2/2011 | EPI_ISL_163970 | clinical sample |
| A/HoChiMinh/1134.2/2011 | EPI_ISL_163969 | clinical sample |
| A/HoChiMinh/1120.2/2011 | EPI_ISL_163968 | clinical sample |
| A/HoChiMinh/1097.2/2011 | EPI_ISL_163967 | clinical sample |
| A/HoChiMinh/1096.2/2011 | EPI_ISL_163966 | clinical sample |
| A/HoChiMinh/1095.2/2011 | EPI_ISL_163965 | clinical sample |
| A/HoChiMinh/1064.1/2011 | EPI_ISL_163964 | clinical sample |
| A/HoChiMinh/1055.1/2011 | EPI_ISL_163963 | clinical sample |
| A/HoChiMinh/1029.12/201 | EPI_ISL_163962 | clinical sample |
| A/HoChiMinh/974.11/2010 | EPI_ISL_163961 | clinical sample |
| A/HoChiMinh/962.11/2010 | EPI_ISL_163960 | clinical sample |
| A/Vietnam/10/2013 | EPI_ISL_145256 | C2/C2 |
| A/Vietnam/3050/20 | EPI_ISL_145097 | C3/C1 |
| A/Vietnam/105/201 | EPI_ISL_145085 | C2/C1 |
| A/Vietnam/56/2011 | EPI_ISL_115091 | C3/C1 |
| A/Vietnam/108/201 | EPI_ISL_115090 | X3/C2 |
| A/Vietnam/57/2010 | \|EPI_ISL_88148 | C3/C1 |
| A/Vietnam/21/2010 | \|EPI_ISL_88147 | C2/C1 |

**Supplementary Table S3.2** Antigenic analysis of A(H3N2) influenza viruses isolated from Ha Nam Cohort participants during the study period

|  | **Virus** |  |  |  |  |  |  |
| --- | --- | --- | --- | --- | --- | --- | --- |
| **Antisera** | Uruguay/716/07* | Perth/16/09* | Hanam/201/09 | Hanam/444/10 | Victoria/361/11 | Hanam/12112/12 | Texas/50/12* |
| Brisbane/10/07 | **640 (ref)** | 40 | 80 | 40 | 160 | 80 | 80 |
| Perth/16/09 | 40 | 160 (ref) | **320** (ref) | 320 | 320 | 320 | 640 |
| Victoria/8/10 | 20 | **320** | 160 | **640** (ref) | 160 | 160 | 1280 |
| Victoria/361/11 | 40 | 160 | 160 | 640 | **640** (ref) | **640** (ref) | 640 |
| Texas/50/12 | 20 | 40 | 80 | 160 | 160 | 160 | 640 (ref) |
| Switz/9715293/13 | 20 | 20 | 80 | 160 | 320 | 320 | 80 |
| HongKong/4801/14* | 40 | 40 | 160 | 160 | 320 | 320 | 160 |
| Michigan/15/14 | 40 | 80 | 160 | 320 | **640** | 320 | **2560** |
| Brisbane/318/16 | 20 | 10 | 40 | 40 | 320 | 160 | 160 |
| Newcastle/30/16 | 40 | 40 | 160 | 320 | **640** | 640 | 160 |
| Victoria/653/17 | 40 | 80 | 160 | 320 | 320 | 640 | 320 |
| Brisbane/34/18 | 10 | 20 | 40 | 40 | 80 | 160 | 40 |

Reference antisera raised against listed strains in rows were tested in HI assay against A(H3N2) virus isolates from the Ha Nam cohort (grey shaded coloum headers) and against adjacent reference viruses. Viruses were propagated in MDCK-SIAT cells or in eggs (* ). Titres that differ more than two-fold compared to each strains reference (ref) antisera are shaded as follows.

**Supplement 4. Participants with evidence of re-infection with the same sub-type**

Forty-two participants exhibited a second 4-fold rise in HI titre against the same virus (15 for H1N1pdm09 and 27 with H3N2Pe09), suggesting re-exposure to or re-infection with an influenza A virus. Examples of these participants are shown in Supplementary Figure S4.1. None of these re-infected participants had illness twice. Some had RTPCR-confirmed illness during one of the infection episodes, but numbers were insufficient to infer whether subclinical infection reduced the risk of ILI upon subsequent infection, or whether ILI reduced the risk of re-infection. Two participants had H3N2Pe09 infection detected by seroconversion without ILI followed, around 3 years later, by infection with RTPCR-confirmed ILI (Supplementary Figure S5.1 B: H005/S01, H171/S03). This equates to an ILI incidence proportion of 7.4% among 27 participants having a second infection, similar to the ILI proportion among all H3N2Pe09 infections (6.7%, Table 1). Two participants had H3N2Pe09 infection with RTPCR-confirmed ILI, followed 3-4 years later by infection without ILI (Supplementary Figure S5.1 B: H145/S01, H150/S01). H3N2Pe09 re-infection rates were similar among participants who were infected during the first two H3N2Pe09 seasons with ILI (16.6%) or without ILI (14.9%). Two participants had H1N1pdm09 infection with ILI followed at least 2.6 Y later by infection without ILI (Supplementary Figure S5.1 A H147/S03, H321/S03). H1N1pdm09 re-infection rates were similar among participants who were infected during the first two H1N1pdm09 seasons with ILI (7.7%) or without ILI (7.4%). Two children had illness twice with alternate influenza virus subtypes (H3N2Pe09 then H1N1pdm09, and vice versa).

**Supplementary Figure S4.1 Observed HI titres among a selection of participants with evidence of re-infection.** Panel A. shows reinfections with H1N1pdm09 for 6/15 participants. Panel B. shows reinfections with H3N2Pe09 for 9/27 participants. For the x-axis, 0 indicates the time of first probable infection. Participants have been selected to show a range of antibody trajectories within the different age groups.

There were no statistically significant differences between those identified as re-infected and those with no evidence of re-infection in terms of observed pre-infection titre, observed starting titre or age (see Supplementary Table S4.1). The GMT of the blood sample taken just prior to re-infection was 15.5 (95%CL: 1.4, 168.5) for H1N1pdm09 and 15.7 (95%CL: 2.6, 92.7) for H3N2pe09. Survival probabilities were calculated using the {survival} package in R. Among those reinfected, the median time to reinfection was 30 months for H1N1pdm09 and 35 months for H3N2Pe09 (Supplementary Figure S4.2).

**Supplementary Table S4.1 Differences in baseline titre, starting titre and age by re-infection status**

|  | Mean (95%CL) | | |  |
| --- | --- | --- | --- | --- |
|  | All participants | Not reinfected | Reinfected | p-val* |
| H1N1pdm09 | (n=227) | (n=212) | (n=15) |  |
| Mean pre-infection titre | 7.4 (4,13.7) | 7.4 (3.9,14) | 7.1 (0.7,76.8) | 0.21 |
| Mean starting titre | 71.3 (38.6,131.7) | 72.3 (38.3,136.3) | 59.2 (5.5,643.5) | 0.15 |
| Mean age | 25.9 (25,26.8) | 25.8 (24.9,26.7) | 26.4 (22.9,29.8) | 0.37 |
| Age group <15y | 82 (35%) | 75 (35%) | 7 (47%) | 0.73 |
| 15-50y | 122 (54%) | 115 (54%) | 7 (47%) |  |
| 50+y | 23 (10%) | 22 (10%) | 1 (7%) |  |
|  |  |  |  |  |
| H3N2Pe09 | (n=211) | (n=184) | (n=27) |  |
| Mean pre-infection titre | 8.3 (4.4,15.8) | 8.3 (4.2,16.5) | 8.2 (1.4,48.8) | 0.39 |
| Mean starting titre | 73.8 (39.1,139.4) | 74.5 (37.7,147.2) | 69.5 (11.7,411) | 0.22 |
| Mean age | 32 (31.1,32.9) | 32.6 (31.6,33.5) | 28.1 (25.5,30.7) | 0.12 |
| Age group <15y | 56 (47%) | 47 (26%) | 9 (33%) | 0.73 |
| 15-50y | 115 (47%) | 99 (54%) | 16 (59%) |  |
| 50+y | 40 (7%) | 38 (21%) | 2 (7%) |  |

Notes: p-value from the Wicoxon rank sum test comparing participants by re-infected status; 95%CL: 95% confidence limits. For Age groups, the p-value is for Fisher’s exact test.

**Supplementary Figure S4.2 Kaplan-Meier plot showing the time to reinfection for 43 individuals with a second 4-fold rise in HI titre.** The time to reinfection was calculated based on the interval between the first and second probable infection dates. Median time to reinfection was 30 months for H1N1pdm09 and 35 months for H3N2Pe09.

For the main decay analysis, all HI titre observations recorded after a sero-conversion were included. In a sensitivity analysis, we excluded the 42 participants who showed evidence of re-infection. Decay curves were fitted on this smaller dataset using the same methods applied in the main analysis. When the models were rerun excluding these participants (189 observations), mean predicted titres for H1N1pdm09 did not drop below the protective threshold, but decreased below a titre of 40 by 8.1 months for H3N2Pe09 (Supplementary Figure S4.3). There were insufficient observations to run the analysis comparing trajectories between those with evidence of infection and those without.


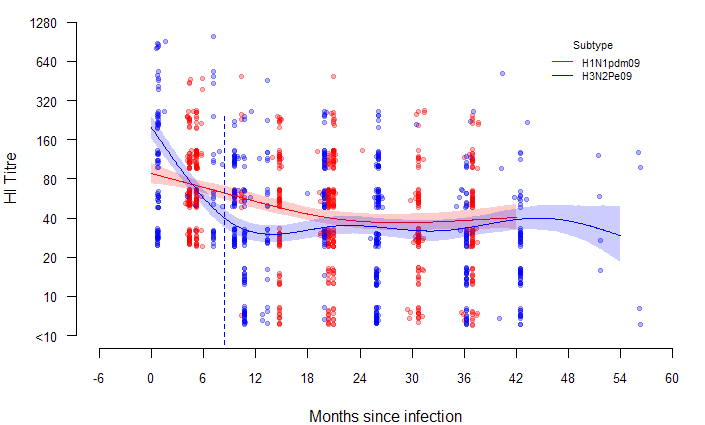


**Supplementary Figure S4.3 HI titre decay when observations from re-infected individuals are removed.** Data are analysed and presented as in Figure 2 after excluding forty-two participants who showed evidence of re-infection to H1N1pdm09 (n=15) or H3N2pe09 (n=27). The dashed line for H3N2pe09 indicates when titres fell below the threshold of 40.

**Supplementary Table S4.2 Sensitivity analysis reporting predicted mean HI titre decay titres and estimated change in titre (absolute and relative) among participants with only one detected infection.**

|  | All infections | | | Excluding participants with more than one infection | | |
| --- | --- | --- | --- | --- | --- | --- |
|  | Predicted mean titre (95%PI) | Δ abs | Δ rel | Predicted mean titre (95%PI) | Δ abs | Δ rel |
| H1N1pdm09 | (n=227) | (n=212) |  |  |  |  |
| Baseline | 79.9 (64,99.7) | 0 | 1 | 87.7 (75,105.9) | 0 | 1 |
| 6m | 68.6 (62.4,75.4) | -11.3 | 1.2 | 68.6 (62.1,75.9) | -19.2 | 1.3 |
| 12m | 55.2 (48.3,62.9) | -24.8 | 1.4 | 53.5 (46.8,61.3) | -34.3 | 1.6 |
| 18m | 42.8 (38.4,47.9) | -37.2 | 1.9 | 42.9 (38.1,48.7) | -44.9 | 2 |
| 24m | 36.8 (32.3,42) | -43.1 | 2.2 | 37.7 (33.1,43.4) | -50 | 2.3 |
| 36m | 40.1 (33.7,47) | -39.8 | 2 | 38.3 (32.2,45.9) | -49.5 | 2.3 |
|  |  |  |  |  |  |  |
| H3N2Pe09 | 176.5 (147.1,217.4) | 0 | 1 | 197.3 (163.5,242.9) | 0 | 1 |
| Baseline | 51.5 (44.7,59.3) | -125 | 3.4 | 56.8 (48.5,66.7) | -140.4 | 3.5 |
| 6m | 29 (25.1,33.1) | -147.6 | 6.1 | 30.8 (26.3,36.2) | -166.4 | 6.4 |
| 12m | 31.7 (27,36.8) | -144.9 | 5.6 | 32.3 (27.3,38.1) | -165 | 6.1 |
| 18m | 34 (29.4,39.5) | -142.5 | 5.2 | 34.9 (30.2,40.7) | -162.4 | 5.7 |
| 24m | 35.9 (30.5,43.1) | -140.7 | 4.9 | 33.4 (27.9,40) | -163.9 | 5.9 |
| 36m | 176.5 (147.1,217.4) | 0 | 1 | 197.3 (163.5,242.9) | 0 | 1 |

Notes: Figures for the re-infected and non-re-infected groups do not add to the totals for all participants as all observations for 4 participants were excluded from the stratified analysis.

**Supplement 5. Titre decay after influenza infection with versus without RTPCR-confirmed illness**

Forty-eight participants with evidence of seroconversion reported an influenza-like illness (ILI), had a swab taken and tested positive to either H1N1pdm09 or H3N2Pe09. Example trajectories for these participants are shown in Supplementary Figure S5.1. In some cases, H3N2-confirmed illness occurred after the first 4-fold rise in antibody titre detected in that individual. Characteristics of participants with seroconversion with and without RTPCR-confirmed illness are compared in Supplementary Table S5.1.

**Supplementary Table S5.1 Differences in baseline titre, starting titre and age by ILI status**

|  | Participants without RTPCR-confirmed illness | Participants with RTPCR-confirmed illness | p-val |
| --- | --- | --- | --- |
| H1N1pdm09 | (n=197) | (n=30) |  |
| Mean pre-infection titre | 7.5 (3.9,14.4) | 7.1 (1.3,39.3) | 0.12 |
| Mean starting titre | 67.4 (34.9,130.3) | 103.1 (19.1,557.1) | 1 |
| Mean Age | 26.5 (25.5,27.4) | 21.9 (19.4,24.3) | 0.04 |
| Age group <15y | 66 (34%) | 16 (53%) | 0.14 |
| 15-50y | 110 (56%) | 12 (40%) |  |
| 50+y | 21 (11%) | 2 (7%) |  |
|  |  |  |  |
| H3N2Pe09 | (n=193) | (n=18) |  |
| Mean pre-infection titre | 8.3 (4.3,16.2) | 8.1 (0.8,81.1) | 0.55 |
| Mean starting titre | 70.2 (36.1,136.4) | 127 (14.4,1120.5) | 0.99 |
| Mean Age | 31.5 (30.5,32.4) | 37.6 (34.4,40.7) | 0.88 |
| Age group <15y | 52 (27%) | 4 (22%) | 0.15 |
| 15-50y | 106 (55%) | 9 (50%) |  |
| 50+y | 35 (18%) | 5 (28%) |  |

Notes: p-value from the Wicoxon rank sum test comparing participants by re-infection status; 95%CL: 95% confidence limits. For Age groups, the p-value is for Fisher’s exact test.

**Supplementary Figure S5.1. Observed HI titres among a selection of participants with RTPCR-confirmed illness.** Panel A. shows RTPCR-confirmed illness events for 9/30 participants with H1N1pdm09. Panel B. shows RTPCR-confirmed illness events associated with H3N2pe09 for 6/18 participants. Along the x-axis, 0 indicates the time of first probable infection. Arrows indicate the date of swab collection for RTPCR-confirmed illness. Participants have been selected to show a range of antibody trajectories within the different age groups. The age of the participant is indicated.

The decay trajectories of participants who reported an RTPCR-confirmed illness were compared with those who did not and are shown in Supplementary Figure S5.2. Participants w RTPCR-confirmed illness tended to have higher titres than those who did not, but their relative change in titre over time was similar (Table S5.2). Models incorporated a product term for RTPCR-confirmed illness status and the time since probable infection; this term did not improve the model’s fit for either subtype.


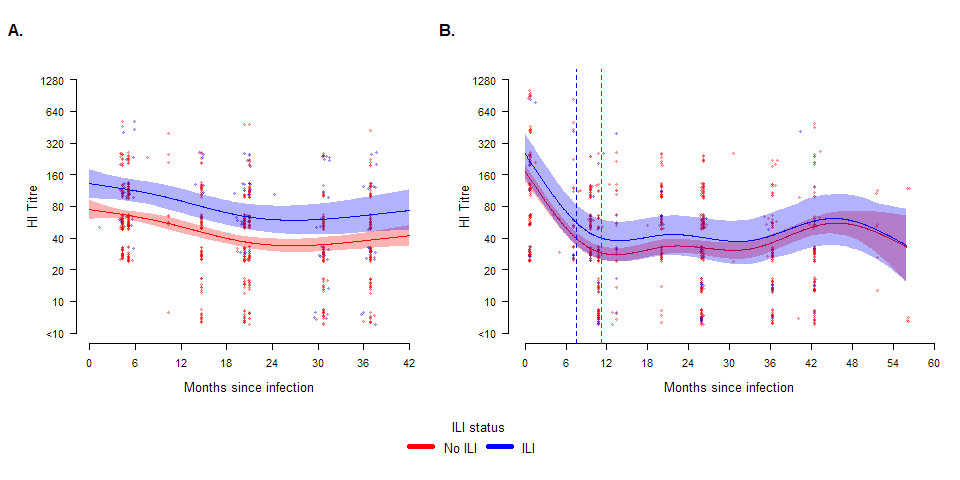


**Supplementary Figure S5.2. Predicted HI titres comparing participants reported an RTPCR-confirmed illness and those who did not.** Panel A. Predicted decay curves for H1N1pdm09. Panel B. Predicted decay curves for H3N2pe09. The two dashed lines for H3 represent when titres were predicted to fall below the threshold.

**Supplementary Table S5.2 Predicted mean HI titre decay titres and estimated change in titre (absolute and relative), by RTPCR-confirmed illness**

|  |  | No RTPCR-confirmed illness | | | RTPCR-confirmed illness | | |
| --- | --- | --- | --- | --- | --- | --- | --- |
|  |  | Predicted mean titre (95%PI) | Δ abs | Δ rel | Predicted mean titre (95%PI) | Δ abs | Δ rel |
| H1N1pdm09 |  | (n=197) | (n=30) |  |  |  |  |
|  | Baseline | 74.6 (60.4,91.1) | 0 | 1 | 131.5 (94,178.4) | 0 | 1 |
|  | 6m | 63.9 (57.9,70.8) | -10.7 | 1.2 | 112.4 (87.2,142) | -19.1 | 1.2 |
|  | 12m | 51.2 (44.6,58.8) | -23.3 | 1.5 | 89.9 (69,117.4) | -41.6 | 1.5 |
|  | 18m | 39.7 (35.2,44.7) | -34.9 | 1.9 | 69.5 (53.1,90.8) | -62 | 1.9 |
|  | 24m | 34.3 (29.8,39.3) | -40.3 | 2.2 | 59.9 (44.7,80.7) | -71.7 | 2.2 |
|  | 36m | 37.6 (31.6,44.5) | -36.9 | 2 | 65.4 (45,96.8) | -66.1 | 2 |
| H3N2Pe09 |  |  |  |  |  |  |  |
|  | Baseline | 170 (140.1,203.3) | 0 | 1 |  | 0 | 1 |
|  | 6m | 50 (43.1,57.4) | -120 | 3.4 | 252.2 (149.5,400) | -180.9 | 3.5 |
|  | 12m | 28.2 (24.3,32.4) | -141.8 | 6 | 71.3 (43.8,107.1) | -213.5 | 6.5 |
|  | 18m | 30.8 (26.2,35.8) | -139.1 | 5.5 | 38.7 (24.3,57.9) | -211.5 | 6.2 |
|  | 24m | 33.3 (28.5,38.4) | -136.7 | 5.1 | 40.7 (25.7,61.2) | -209.9 | 6 |
|  | 36m | 35.6 (29.3,42.7) | -134.4 | 4.8 | 42.3 (26.6,63.8) | -210.4 | 6 |
